# Supplementary material for: Investigation of an Impedimetric LaSrMnO3-Au/Y2O3-ZrO2-Al2O3 Composite NOx Sensor
Source: Materials (Basel). 2022 Feb 2;15(3):1165. doi: 10.3390/ma15031165 (PMC8837989; doi:10.3390/ma15031165)
Supplement: Supplementary file 1 [file materials-15-01165-s001.zip › materials-1570515-supplementary.pdf]

Supplementary Materials

Investigation of an Impedimetric $LaSrMnO_3$-Au/Y_2O_3-ZrO_2-Al_2O_3 Composite NO_x Sensor

Nabamita Pal *,†, Gaurab Dutta *,†, Khawlah Kharashi and Erica P. Murray *

Institute for Micromanufacturing, Louisiana Tech University, Ruston, LA 71272, USA; omsoosoo2010@hotmail.com

* Correspondence: nabamitapal84@gmail.com (N.P.); dutta.gaurab@gmail.com (G.D.); emurray@latech.edu (E.P.M.); Tel.: +1-318-497-3752 (N.P.); +1-318-497-3751 (G.D.); +1-318-257-5148 (E.P.M.)

† These authors contributed equally to this work.

Section S1 Elemental Mapping (EDS) of $LaSrMnO_3$-Au Composite Electrode.

Figure S1 and ***Error! Reference source not found.*** show the atomic percent and mole content of the LSM-Au sensing electrode elements. The EDS measurements confirmed the electrode elemental composition; however, the analysis of the elemental concentration deviated from the expected values. A likely reason is that these complex heterogenous oxides, such as LSM with non-stoichiometric phases with enriched Mn and depleted La, Sr elements have overlapping peaks involving oxygen that can interfere with accurate detection [1]. Such deviation in measurements can arise from the background signal resulting in low detection of elements and strong matrix effects from multiple electrons scattering in the thick LSM-Au sample. Such scattering may cause a reduction in the spatial resolution and chemical sensitivity of LSM-Au samples during EDS measurements. Since the LSM-Au electrode cross-section was non-homogenous and EDS captured only a portion of the cross-section, the measurements for Au were lower than the actual amount of Au within the entire bulk electrode.

Nonetheless, the EDS measurements enabled an analysis of the elemental composition and estimates of the proportion of such elements at different positions within the LSM-Au electrode. The elemental measurements consequently provided localized mapping of the bulk LSM-Au sample. The data for La signified ~0.4% of the lattice volume sites had La^{3+} cation deficiencies, which aided Mn^{3+} ion ($3d^3$ electronic configuration) enhancement activity [2–5]. Though the stoichiometric ratio (A/B>1) for (La,Sr)MnO_3 may have possessed other small proportions of low conductive non-stoichiometric phases such as $(La,Sr)_3Mn_2O_7$, La_2O_3, and $(La,Sr)_2Mn_2O_4$ promoted by Sr doping, it did not contribute towards sluggishness during charge transfer [6]. Increased Sr doping may result in the distributed partial oxidation of Mn ions to form the formal $(3+\delta)^+$ oxidation state in LSM leads to shrinking Mn-O bonds, thus decreasing unit-cell volume [7–10]. The $La_{0.8}Sr_{0.2}MnO_3$ usually has monoclinic symmetry in the lattice structure but does possess hexagonal-rhombohedral distortions in the lattice structure for Sr doping range $0.1<x<0.5$ [6]. The minor phases such as, $(La, Sr)_2Mn_2O_4$ are only stable and observable at lower temperatures ~ 1200°C for Sr doping level (x)> ~ 0.3 mole [4,6]. We assume $(La, Sr)_2Mn_2O_4$ phases would not be adversely affecting the LSM-Au surface response after exposure around 1400°C [4,6]. The $(La, Sr)_3Mn_2O_7$ minor phases (Ruddlesden-Popper phase) may be present at a temperature around 1400°C only for the higher Sr doping levels (x>0.3)[4]. The A-site nonstoichiometry of LSM at the electrode surface facilitated more cation vacancies via Schottky disorder, which promoted charge transfer reactions at the electrode-electrolyte interface [3].

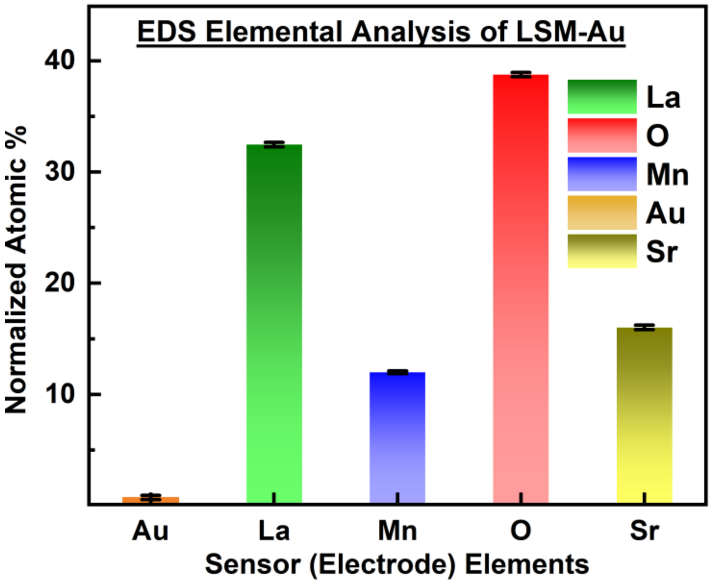

Figure S1 The figure shows the atomic percentage of LSM-Au governing the phase behavior within the sensor.

Table S1. EDS analysis for LSM-Au.

Elements	At%±Error	Mole±Error
Lanthanum (La)	32.5±1.5	0.44±0.02
Strontium (Sr)	16±1.6	0.22±0.02
Manganese (Mn)	12±0.4	0.16±0.00
Oxygen (O)	38.6±2.3	0.53±0.05
Gold (Au)	0.74±0.1	0.01±0.00

*All error measurements are approximated based on the number of measurements at four different electrode sample sites.

Section S2 Structural analysis via XRD of FSZ-PSZ-2wt%Al_2O_3/$LaSrMnO_3$-Au Sensor.

Further analysis of the composite electrolyte and electrode structure is provided by considering the associated space group, which describes the crystal orientation that influenced defects within material phases. The XRD data in Figure 3b indicated PSZ (4.7 mol% Y_2O_3-ZrO_2)contained a mixture of both tetragonal and monoclinic phases of ZrO_2 (monoclinic-ZrO_2,JCPDS: 37-1484)(tetragonal-ZrO_2, JCPDS: 42-1164), which related to the space group of $P4_2/nmc$ & $P2_1/c$, respectively [11–14]. In addition, the average crystallite size obtained for PSZ was about 46 nm. Figure 3c shows FSZ (8 mol% Y_2O_3-ZrO_2) primarily consisted of distorted cubic zirconia phases (cubic-ZrO_2, JCPDS: 49-1642) due to having a space group of $Fm\bar{3}m$. The average crystallite size of FSZ was approximately 3 nm, which has been seen in other studies [11,15–18]. The crystal planes and space groups were indicative of defects and arrangements that corresponded to the electrical and mechanical properties of the electrolyte.

The LSM diffraction peaks were indexed to the pseudo-cubic perovskite structure (space group $Pm\bar{3}m$)with high crystallinity [19,20]. Moreover, splitting of the (220) diffraction peak into two peaks coupled with the (224) diffraction peak attributed to the tetragonal crystal structure (space group 14/mcm), resulting in the formation of tetragonal polymorph for LSM [21,22]. The XRD finding indicated that the nanoparticles in LSM and LSM-Au samples might be a mixture of cubic and tetragonal structures. The

average crystallite size of LSM was about 10 nm. The Sr doping changed the valence state of Mn ions ($Mn^{3+} \rightarrow Mn^{4+}$) to keep both samples electrically neutral. The presence of tetragonal phases exerted less distortion or irregularities in both samples' crystal structure, which helped enlarge the conduction band. Such enlargement enhanced the double exchange phenomenon; thereby, enhancing the charge transfer properties at a lower frequency [23].

Section S3

Table S1. XPS analysis for $LaSrMnO_3$-Au / FSZ Composite NO_x Sensor in Table S2.

**FSZ composite electrolyte**									
Spectra	Zr3d & Y3d								Al2s
	Spin doublet Splits								
	Monoclinic phases of Zirconia		Tetragonal/cubic phases of Zirconia		Tetragonal phases of Yttria		Cubic phases of Yttria		α-Al_2O_3
	Zr3d$_{5/2}$	Zr3d$_{3/2}$	Zr3d$_{5/2}$	Zr3d$_{3/2}$	Y3d$_{5/2}$	Y3d$_{3/2}$	Y3d$_{5/2}$	Y3d$_{3/2}$	Al2s
BE (eV)	181.2	183.5	182.0	184.5	156.6	158.7	157.2	159.3	119.3
At%	13	13	29.4	29.4	*3.2*	3.2	4.4	4.4	100
FWHM (eV)	1.82	2.14	1.35	1.42	1.70	1.70	1.25	1.35	2.76

**LSM-Au electrode**											
Spectra	La3d							Sr3d			
	Spin doublet Splits						Satellite	Spin doublet Splits			
	Electron transfer from O_2 ligand		From La_2O_3 of the inner LSM-Au surface		Shake up features from outer LSM-Au surface		Mixing of $3d^94f^2$ & $3d^94f^3$ states	Sr^{2+} ions in the LSM lattice		SrO species on the LSM	
	La3d$_{5/2}$	La3d$_{3/2}$	La3d$_{5/2}$	La3d$_{3/2}$	La3d$_{5/2}$	La3d$_{3/2}$	La3d	Sr3d$_{5/2}$	Sr3d$_{3/2}$	Sr3d$_{5/2}$	Sr3d$_{3/2}$
BE (eV)	833.9	850.8	835.3	852	838.4	855.2	848	132.5	134.3	133.0	134.8
At%	10.6	10.6	18.4	18.3	21	20.9	0.2	31.6	31.6	18.4	18.4
FWHM (eV)	1.48	1.52	2.76	3.11	2.33	2.86	5.55	0.81	0.92	1.85	3.50

Spectra	Mn2p				Au4f		Mn3s	
	Spin doublet Splits				Doublet Split		Relative Mn^{4+} content	
	$LaMn^{3+}O_3$ species of LSM-Au		$SrMn^{4+}O_3$ species of LSM-Au		Metallic Au^0 from LSM-Au surface		High Spin	Low Spin
	Mn2p$_{3/2}$	Mn2p$_{1/2}$	Mn2p$_{3/2}$	Mn2p$_{1/2}$	Au4f$_{7/2}$	Au4f$_{5/2}$	Mn3s$_1$	Mn3s$_2$
BE (eV)	641.5	653.2	644.1	655.3	84.3	87.9	82.4	90.6
At%	42	42	8	8	5.5	5.5	53.2	35.8
FWHM (eV)	2.92	3.23	2.59	2.47	2.67	2.44	2.07	2.73

Section S4 Defect Model Influencing Ion Transport in FSZ Composite Electrolyte

According to the Kroeger-Vink defect model, the porous FSZ composite electrolyte results in oxygen vacancies for charge compensation that helps to achieve optimum conductivity (see Figure. 5a) [24,25].

$$Y_2O_3 \xrightarrow{ZrO_2} 2Y'_{Zr} + V''_{\ddot{o}} + O_o^x \quad (1)$$

Here, the Y_{Zr}' is the Y^{3+} impurity in the site of Zr^{4+} in the lattice having a relative charge of -1, the $V\ddot{o}''$ represents the free oxygen vacancy with a relative charge of +2, and the O_o^x denotes the quasi-stable lattice oxygen vacancy generated under reducing conditions. The O_o^x undergoes through a Schottky defect pair formation reaction under equilibrium condition releasing oxygen from the FSZ-PSZ-2wt% Al_2O_3 lattice & $V\ddot{o}''$.

$$O_o^x \leftrightarrow \frac{1}{2}O_{2(g)} + V''_{\ddot{o}} + 2e^- \quad (2)$$

During the electroneutrality condition (having a proportional relation or equilibrium between oxygen vacancies and holes) at nominal oxygen partial pressure, P_{O2} , the overall oxygen vacancy $V\ddot{o}''$is dominated by Y_{Zr}', the Y^{3+} impurity and electron concentration are as follows [24].

$$2[V''_{\ddot{o}}] \leftrightarrow [Y'_{Zr}] + [e^-] \quad (3)$$

The oxygen ion diffusion in FSZ composite electrolyte occurred via vacancy-ion interactions to the triple-phase-boundary (TPB). The ion diffusion was regulated by a vacancy hopping mechanism, mostly at cation-bounded lattice edges in the FSZ composite electrolyte [24,26,27].

Section S5 Electrochemical Reaction Pathways and Heterogeneous Diffusion During NO_x Sensing at Electrolyte-Electrode Interface of Composite NO_x Sensor.

The Bode Plot provides an additional means of analyzing the electrochemical behavior of the FSZ composite sensor, as it displays the frequencies associated with the electrical response with and without NO_x presence. Figure. **S2** shows the Bode plot of the FSZ composite sensor that featured Hockey-Stick (10 to 10^6 Hz) and Ski-slope (1 to 10^4 Hz) shapes, indicating the sophisticated nature of charge transfer occurring via multiple LSM-Au surface pathways [9,28]. The Hockey-Stick shape was symbolic of the low impedance of the FSZ composite electrolyte material, where the spectra almost flattened out at higher frequency. The constant phase element Q_{HF} represented the high-frequency dielectric dispersive capacitance. The Q_{HF} represents the coupling current leakage through the FSZ composite porous electrolyte. The electrolyte resistance was the following flat part of the dielectric at the high-frequency region modulated by the surface geometry of the electrolyte, including thickness and the effective impedance of the gas diffusion path offered by the FSZ composite electrolyte. The Q_{HF} and electrolyte resistance, R_{HF} do not differ with and without 100 ppm NO_x, suggesting no cross-reaction resulting in changed current leakage.

The n values associated with the Q_{HF} were around 0.75 for data collected with and without 100 ppm NO_x. The n values show the non-ideal capacitive nature of the dielectric coupling at the high frequency [23]. During 100 ppm NO_x exposure, the electrode or interface capacitance value decreased due to a lower impedance pathway for enhanced charge transfer. The Ski-slope shape indicated a parasitic leakage pathway, resulting in a sizeable actual impedance without 100 ppm NO_x. The lower frequency region (<10 Hz) indicated the parasitic leakage associated with the reaction pathways through the grain boundary and associated Au particles at the LSM surface. The decrease in the magnitude of the impedance, |Z|, at 1 Hz signified a leakage path due to shunt resistance R_{LF}. The parasitic pathway occurred at a high impedance region that offered significantly higher impedance without 100 ppm NO_x and enhanced charge transfer at the LSM surface in the presence of 100 ppm NO_x. During exposure to 100 ppm NO_x, the lower frequency curve flattens out more, signified the availability of a comparably lower impedance path, revealing a more

resistive nature [28,29]. The PSZ mostly had prevailing tetragonal phases with a mix of cubic phases and FSZ composed of cubic phases, both particle sizes ranging from 40-1000 nm [12]. The addition of PSZ combined with Al_2O_3 in FSZ provided better fracture toughness to the composite electrolyte but resulted in lower electrical conductivity at a higher temperature (T>550°C).

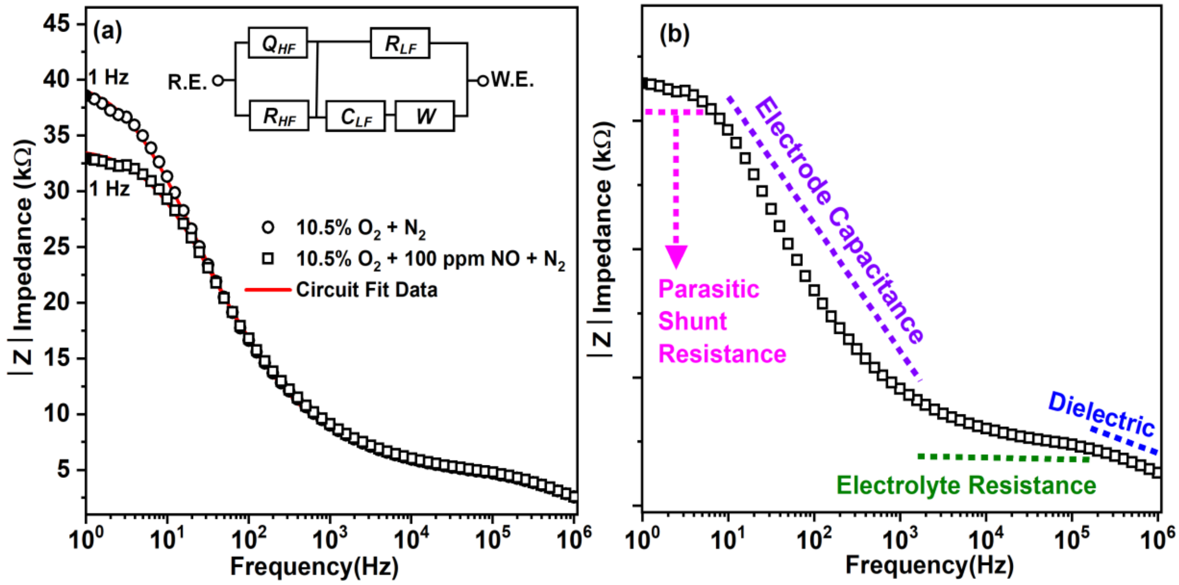

Figure. S2 (**a**) The bode Impedance plot of the FSZ composite NO_x sensor at 575°C with and without 100 ppm NO present. The Bode plot comprises varied shapes like Hockey-Stick (10 to 10^6 Hz) and Ski-slope (1 to 10^4 Hz). Inset the equivalent represents both types of NO_x sensors. (**b**) The high-frequency region comprises the coupling of electrolyte resistance through a dielectric constant phase element (CPE). The lower frequency region is composed of electrode capacitance and parasitic shunt resistance.

Section S6

Table S3. for R^2 Values Obtained for Linear Fitting Trends Obtained from Figure. 8 For both Dry and Humidified Conditions in FSZ and FSZ Composite Sensor.

	FSZ Composite NO_x Sensor				FSZ NO_x Sensor			
Range	0-25 ppm		25-100 ppm		0-50 ppm		50-100 ppm	
Conditions	Dry	Humid	Dry	Humid	Dry	Humid	Dry	Humid
R^2	0.98534	0.97617	0.99595	0.99951	0.99952	0.99992	0.99936	0.99985

Note: Dry condition is without 10% H_2O; the wet condition is with 10% H_2O. Besides, both conditions have 10.5 % O_2, NO, N_2 present during measurement. The R^2 values confirm the linearity of the presented dataset in Figure. 8. All Linear Fitting is performed in OriginPro Software 2021b.

Section S7 Water Cross Sensitivity for FSZ Composite NO_x Sensor at Various Temperatures.

The water cross-sensitivity diminished with increasing operating temperatures beyond (T>575°C) for the FSZ composite NO_x sensor. The water cross-sensitivity demonstrated a decreasing trend of ~7% as the operating temperature increases by 25°C from 575 to 600°C (Figure S3).

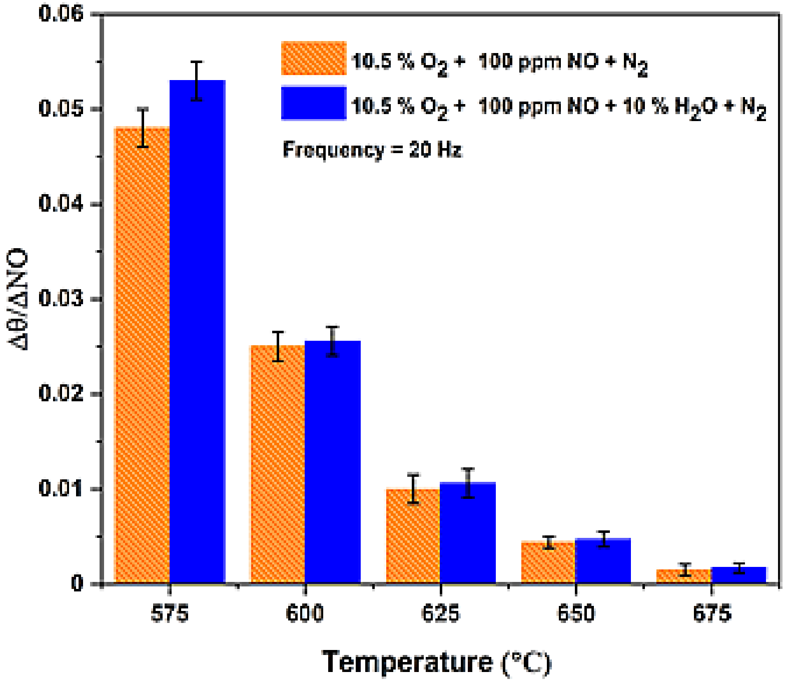

Figure S3 Change in water cross-sensitivity with increasing operating temperature for FSZ composite NO_x sensor.

Section S8 Rate Limiting Mechanism Associated with FSZ Composite NO_x Sensor.

The rate-limiting mechanisms associated with the FSZ composite sensors were evaluated using the power-law relationship $R_{LF} \propto P_{O2}{}^{m}$ where R_{LF} was the charge transfer resistance determined from equivalent circuit analysis of the low-frequency arc, P_{O2} described the oxygen partial pressure, and m indicated the rate-limiting mechanism. Figure S4 shows the value of the power-law exponent, m, for the FSZ composite sensors. The transport of partially reduced atomic oxygen to the TPB where charge transfer occurs has been associated with a slope value of m = - 0.25 [30,31]. The P_{O2} dependence for dry gas conditions resulted in data extremely near this value, thus suggesting atomic oxygen reduction was the rate-limiting mechanism for the FSZ composite sensors. The sensing LSM-Au electrode surface may act as an oxygen-deficient surface at low oxygen partial pressures [3,9,10]. Such a scenario likely affected the mechanism of surface oxygen vacancy formation in the FSZ composite electrolyte. The increase in the P_{O2} dependence due to the addition of 10% water in the operating environment suggests that charge transfer became more rapid as partial reduction of atomic oxygen was enhanced.

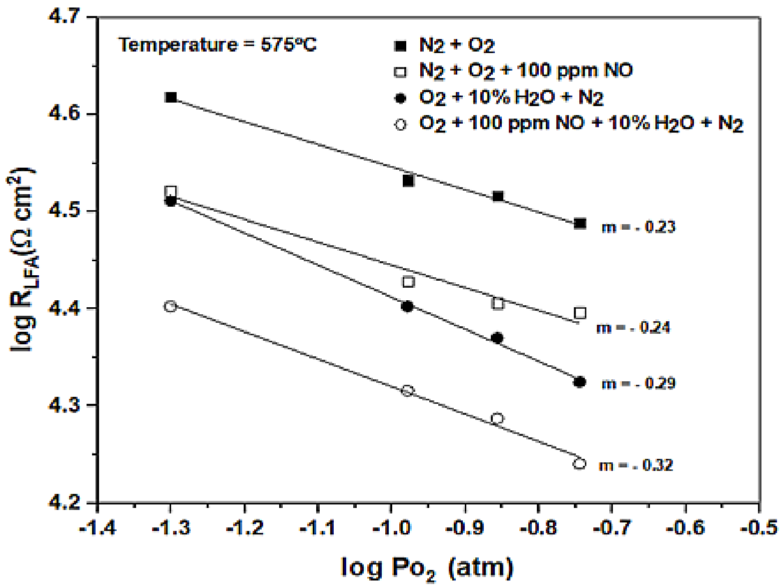

Figure S4 Power law exponent determination for FSZ composite sensors with various gas concentrations of NO and H_2O at 575°C.

References

1. Y. Yu, J. Liu, H.O. Finklea, H. Abernathy, P.R. Ohodnicki, T. Kalapos, G.A. Hackett, Chemical Analysis of Activation Process of LSM Thin Film Electrode, ECS Trans. 78 (2017) 701. https://doi.org/10.1149/07801.0701ecst.
2. M. Pavone, A.B. Muñoz-García, A.M. Ritzmann, E.A. Carter, First-Principles Study of Lanthanum Strontium Manganite: Insights into Electronic Structure and Oxygen Vacancy Formation, J. Phys. Chem. C. 118 (2014) 13346–13356. https://doi.org/10.1021/jp500352h.
3. S.P. Jiang, Development of lanthanum strontium manganite perovskite cathode materials of solid oxide fuel cells: a review, J. Mater. Sci. 43 (2008) 6799–6833. https://doi.org/10.1007/s10853-008-2966-6.
4. F. Zheng, L.R. Pederson, Thermodynamic Properties of Sr-Doped $LaMnO_3$ Perovskite in the La-Sr-Mn-O System, J. Electrochem. Soc. 146 (1999) 2817–2820. https://doi.org/10.1149/1.1392013.
5. Y.J. Leng, S.H. Chan, K.A. Khor, S.P. Jiang, Development of LSM/YSZ composite cathode for anode-supported solid oxide fuel cells, J. Appl. Electrochem. 34 (2004) 409–415. https://doi.org/10.1023/B:JACH.0000016627.29374.24.
6. F. Zheng, L.R. Pederson, Phase Behavior of Lanthanum Strontium Manganites, J. Electrochem. Soc. 146 (1999) 2810. https://doi.org/10.1149/1.1392012.
7. S. Raz, K. Sasaki, J. Maier, I. Riess, Characterization of adsorbed water layers on Y_2O_3-doped ZrO_2, Solid State Ion. 143 (2001) 181–204. https://doi.org/10.1016/S0167-2738(01)00826-8.
8. S. Zhuiykov, An investigation of conductivity, microstructure and stability of HfO_2–ZrO_2–Y_2O_3–Al_2O_3 electrolyte compositions for high-temperature oxygen measurement, J. Eur. Ceram. Soc. 20 (2000) 967–976. https://doi.org/10.1016/S0955-2219(99)00233-2.

.9 T. Yang, J. Liu, Y. Yu, Y.-L. Lee, H. Finklea, X. Liu, H. W. Abernathy, G. A. Hackett, Modeling of the oxygen reduction reaction for dense LSM thin films, Phys. Chem. Phys. 19 (2017) 30464–30472. https://doi.org/10.1039/C7CP05899C.

10. D. Lee, J. Tan, K.H. Chae, B. Jeong, A. Soon, S.-J. Ahn, J. Kim, J. Moon, Chemically Driven Enhancement of Oxygen Reduction Electrocatalysis in Supported Perovskite Oxides, J. Phys. Chem. Lett. 8 (2017) 235–242. https://doi.org/10.1021/acs.jpclett.6b02503.
11. K. Li, J. Chen, J. Peng, S. Koppala, M. Omran, G. Chen, One-step preparation of CaO-doped partially stabilized zirconia from fused zirconia, Ceram. Int. 46 (2020) 6484–6490. https://doi.org/10.1016/j.ceramint.2019.11.129.
12. F. Zhang, B. Van Meerbeek, J. Vleugels, Importance of tetragonal phase in high-translucent partially stabilized zirconia for dental restorations, Dent. Mater. 36 (2020) 491–500. https://doi.org/10.1016/j.dental.2020.01.017.

13. M.A. Gafur, Md.S.R. Sarker, Md.Z. Alam, M.R. Qadir, Effect of 3 mol% Yttria Stabilized Zirconia Addition on Structural and Mechanical Properties of Alumina-Zirconia Composites, Mater. Sci. Appl. 08 (2017) 584–602. https://doi.org/10.4236/msa.2017.87041.
14. N. Ishizawa, A. Saiki, T. Yagi, N. Mizutani, M. Kato, Twin-Related Tetragonal Variants in Yttria Partially Stabilized Zirconia, J. Am. Ceram. Soc. 69 (1986) C-18-C–20. https://doi.org/10.1111/j.1151-2916.1986.tb04724.x.
15. W. Zhu, S. Nakashima, E. Marin, H. Gu, G. Pezzotti, Microscopic mapping of dopant content and its link to the structural and thermal stability of yttria-stabilized zirconia polycrystals, J. Mater. Sci. 55 (2020) 524–534. https://doi.org/10.1007/s10853-019-04080-9.
16. A.F. Al-Attar, S. B. H. Farid, F. A. Hashim, Characterizations of Synthetic 8mol% YSZ with Comparison to 3mol %YSZ for HT-SOFC, Eng. Technol. J. 38 (2020) 491–500. https://doi.org/10.30684/etj.v38i4A.351.
17. B. Xing, C. Cao, W. Zhao, M. Shen, C. Wang, Z. Zhao, Dense 8 mol% yttria-stabilized zirconia electrolyte by DLP stereolithography, J. Eur. Ceram. Soc. 40 (2020) 1418–1423. https://doi.org/10.1016/j.jeurceramsoc.2019.09.045.
18. G.J. la O', B. Yildiz, S. McEuen, Y. Shao-Horn, Probing Oxygen Reduction Reaction Kinetics of Sr-Doped $LaMnO_3$ Supported on Y_2O_3-Stabilized ZrO_2 : EIS of Dense, Thin-Film Microelectrodes, J. Electrochem. Soc. 154 (2007) B427. https://doi.org/10.1149/1.2508887.
19. K. Huang, X. Chu, W. Feng, C. Zhou, W. Si, X. Wu, L. Yuan, S. Feng, Catalytic behavior of electrospinning synthesized $La_{0.75}Sr_{0.25}MnO_3$ nanofibers in the oxidation of CO and CH_4, Chem. Eng. J. 244 (2014) 27–32. https://doi.org/10.1016/j.cej.2014.01.056.
20. Y.W. Duan, X.L. Kou, J.G. Li, Size dependence of structure and magnetic properties of $La_{0.7}Sr_{0.3}MnO_3$ nanoparticles, Phys. B Condens. Matter. 355 (2005) 250–254. https://doi.org/10.1016/j.physb.2004.10.100.
21. H. Arandiyan, H. Dai, J. Deng, Y. Wang, H. Sun, S. Xie, B. Bai, Y. Liu, K. Ji, J. Li, Three-Dimensionally Ordered Macroporous $La_{0.6}$ $Sr_{0.4}$ MnO_3 Supported Ag Nanoparticles for the Combustion of Methane, J. Phys. Chem. C. 118 (2014) 14913–14928. https://doi.org/10.1021/jp502256t.
22. P.M. Woodward, T. Vogt, D.E. Cox, A. Arulraj, C.N.R. Rao, P. Karen, A.K. Cheetham, Influence of Cation Size on the Structural Features of $Ln_{1/2}$ $A_{1/2}$ MnO_3 Perovskites at Room Temperature, Chem. Mater. 10 (1998) 3652–3665. https://doi.org/10.1021/cm980397u.
23. K. Yan, R. Fan, M. Chen, K. Sun, L. Yin, H. Li, S. Pan, M. Yu, Perovskite (La, Sr)MnO_3 with tunable electrical properties by the Sr-doping effect, J. Alloys Compd. 628 (2015) 429–432. https://doi.org/10.1016/j.jallcom.2014.12.137.
24. M.T. Elm, J.D. Hofmann, C. Suchomski, J. Janek, T. Brezesinski, Ionic Conductivity of Mesostructured Yttria-Stabilized Zirconia Thin Films with Cubic Pore Symmetry—On the Influence of Water on the Surface Oxygen Ion Transport, ACS Appl. Mater. Interfaces. 7 (2015) 11792–11801. https://doi.org/10.1021/acsami.5b01001.
25. S. Killa, L. Cui, E. Murray, D. Mainardi, Kinetics of Nitric Oxide and Oxygen Gases on Porous Y-Stabilized ZrO_2-Based Sensors, Molecules. 18 (2013) 9901–9918. https://doi.org/10.3390/molecules18089901.
26. J.-H. Park, R.N. Blumenthal, Electronic Transport in 8 Mole Percent Y_2O_3 - ZrO_2, J. Electrochem. Soc. 136 (1989) 2867. https://doi.org/10.1149/1.2096302.
27. H. Ding, A.V. Virkar, F. Liu, Defect configuration and phase stability of cubic versus tetragonal yttria-stabilized zirconia, Solid State Ion. 215 (2012) 16–23. https://doi.org/10.1016/j.ssi.2012.03.014.
28. M. Straka, B. Shafer, S. Vasudevan, C. Welle, L. Rieth, Characterizing Longitudinal Changes in the Impedance Spectra of In-Vivo Peripheral Nerve Electrodes, Micromachines. 9 (2018) 587. https://doi.org/10.3390/mi9110587.
29. Y. Gönüllü, K. Kelm, S. Mathur, B. Saruhan, Equivalent Circuit Models for Determination of the Relation between the Sensing Behavior and Properties of Undoped/Cr Doped TiO_2 NTs, Chemosensors. 2 (2014) 69–84. https://doi.org/10.3390/chemosensors2010069.
30. Y. Takeda, R. Kanno, M. Noda, Y. Tomida, O. Yamamoto, Cathodic Polarization Phenomena of Perovskite Oxide Electrodes with Stabilized Zirconia, J. Electrochem. Soc. 134 (1987) 2656–2661. https://doi.org/10.1149/1.2100267.
31. L. Navarrete, C. Solís, J.M. Serra, Boosting the oxygen reduction reaction mechanisms in IT-SOFC cathodes by catalytic functionalization, J. Mater. Chem. A. 3 (2015) 16440–16444. https://doi.org/10.1039/C5TA05187H.
